# Impaired Insulin Signaling is Associated with Hepatic Mitochondrial Dysfunction in IR^+/−^-IRS-1^+/−^ Double Heterozygous (IR-IRS1dh) Mice

**DOI:** 10.3390/ijms18061156

**Published:** 2017-05-30

**Authors:** Andras Franko, Alexander Kunze, Marlen Böse, Jürgen-Christoph von Kleist-Retzow, Mats Paulsson, Ursula Hartmann, Rudolf J. Wiesner

**Affiliations:** 1 Institute of Vegetative Physiology, Medical Faculty, University of Köln, Robert-Koch-Str. 39, D-50931 Cologne, Germany; marlen.b@gmx.net (M.B.); juergen-christoph.von-kleist-retzow@uk-koeln.de (J.-C.v.K.-R.); rudolf.wiesner@uni-koeln.de (R.J.W.); 2 Department of Internal Medicine IV, Division of Endocrinology, Diabetology, Angiology, Nephrology and Clinical Chemistry, University Hospital Tübingen, Otfried-Müller-Str. 10, D-72076 Tübingen, Germany; 3 Center for Biochemistry, Medical Faculty, University of Köln, Joseph-Stelzmann-Str. 52, D-50931 Cologne, Germany; alexander-kunze@gmx.net (A.K.); mats.paulsson@uni-koeln.de (M.P.); ursula.hartmann@uni-koeln.de (U.H.); 4 Department of Pediatrics, University Hospital Cologne, Kerpener Str. 62, D-50937 Cologne, Germany; 5 Center for Molecular Medicine Cologne (CMMC), University of Köln, D-50931 Cologne, Germany; 6 Cologne Excellence Cluster on Cellular Stress Responses in Ageing-associated Diseases (CECAD), University of Köln, D-50931 Cologne, Germany

**Keywords:** insulin signaling, insulin receptor, insulin receptor substrate 1, mitochondria, liver, skeletal muscle, glucose metabolism

## Abstract

Mitochondria play a pivotal role in energy metabolism, but whether insulin signaling per se could regulate mitochondrial function has not been identified yet. To investigate whether mitochondrial function is regulated by insulin signaling, we analyzed muscle and liver of insulin receptor (IR)^+/−^-insulin receptor substrate-1 (IRS-1)^+/−^ double heterozygous (IR-IRS1dh) mice, a well described model for insulin resistance. IR-IRS1dh mice were studied at the age of 6 and 12 months and glucose metabolism was determined by glucose and insulin tolerance tests. Mitochondrial enzyme activities, oxygen consumption, and membrane potential were assessed using spectrophotometric, respirometric, and proton motive force analysis, respectively. IR-IRS1dh mice showed elevated serum insulin levels. Hepatic mitochondrial oxygen consumption was reduced in IR-IRS1dh animals at 12 months of age. Furthermore, 6-month-old IR-IRS1dh mice demonstrated enhanced mitochondrial respiration in skeletal muscle, but a tendency of impaired glucose tolerance. On the other hand, 12-month-old IR-IRS1dh mice showed improved glucose tolerance, but normal muscle mitochondrial function. Our data revealed that deficiency in IR/IRS-1 resulted in normal or even elevated skeletal muscle, but impaired hepatic mitochondrial function, suggesting a direct cross-talk between insulin signaling and mitochondria in the liver.

## 1. Introduction

Since mitochondria have a central role in cellular metabolism, there is dynamic and continuous crosstalk between them and other organelles like nucleus, endoplasmic reticulum (ER), and cell membrane, using metabolites like ATP, NAD(P)H, reactive oxygen species (ROS), tricarboxylic cycle (TCA) metabolites as well as calcium as signaling molecules [1]. Mitochondrial dysfunction is implicated in many diseases and there is increasing evidence that mitochondrial function in skeletal muscle or liver is altered in insulin resistance and diabetes [2,3]. On the other hand, little knowledge has been established on the direct crosstalk between insulin signaling and mitochondria.

In our previous studies, we already investigated the interaction between insulin resistance and mitochondrial function in different tissues like muscle and liver in various animal models. We found that the ablation of insulin receptor in muscle (MIRKO mice) or insulin deficiency caused by beta-cell reduction (streptozotocin (STZ) treated mice) led to an impairment of skeletal muscle mitochondrial performance [4]. In contrast to muscle, liver mitochondria of insulin-deficient STZ mice showed better coupling and elevated oxygen consumption [5], suggesting a compensatory capacity of liver in insulin-deficient states [6].

Insulin receptor (IR) and insulin receptor substrate-1 (IRS-1) are the first two key molecules transmitting insulin signaling to the cell [7]. Several mutations or polymorphisms have been shown in their genes, which were associated with insulin resistance and type 2 diabetes [7]. Mouse models serve as a unique tool to investigate the consequence of insulin resistance due to ablation of these receptor types in specific tissues (IR knockout (KO) mice) [8]. These mice have been intensively studied earlier and most of IR KO mice showed disturbed glucose metabolism [8], however, there is limited information about mitochondrial function. Our aim was to study both liver and skeletal muscle mitochondrial function in a well-established mouse model of insulin resistance, IR^+/−^-IRS-1^+/−^ double heterozygous (IR-IRS1dh) mice [9], at advanced age, since moderately reduced insulin signaling was implicated in increased lifespan [10] and these mice have been rarely analyzed at older age [9,11,12]. IR-IRS1dh mice were originally generated by the Kahn laboratory and are characterized by attenuated hepatic and skeletal muscle insulin signaling [9,12]. In order to study (i) whether moderately impaired insulin signaling could affect mitochondrial function in different tissues and (ii) whether ageing could impact glucose metabolism or mitochondrial function, we have analyzed mitochondrial performance in hepatic and skeletal muscle mitochondria of IR-IRS1dh mice at 6 and 12 months of age.

## 2. Results

### 2.1. IR^+/−^-IRS-1^+/−^ Double Heterozygous (IR-IRS1dh) Mice

IR-IRS1dh mice demonstrated less body weight and decreased fasting blood glucose levels at both ages (6 and 12 months old) compared to age-matched wild-type (wt) controls (Table 1).

**Figure 1:**
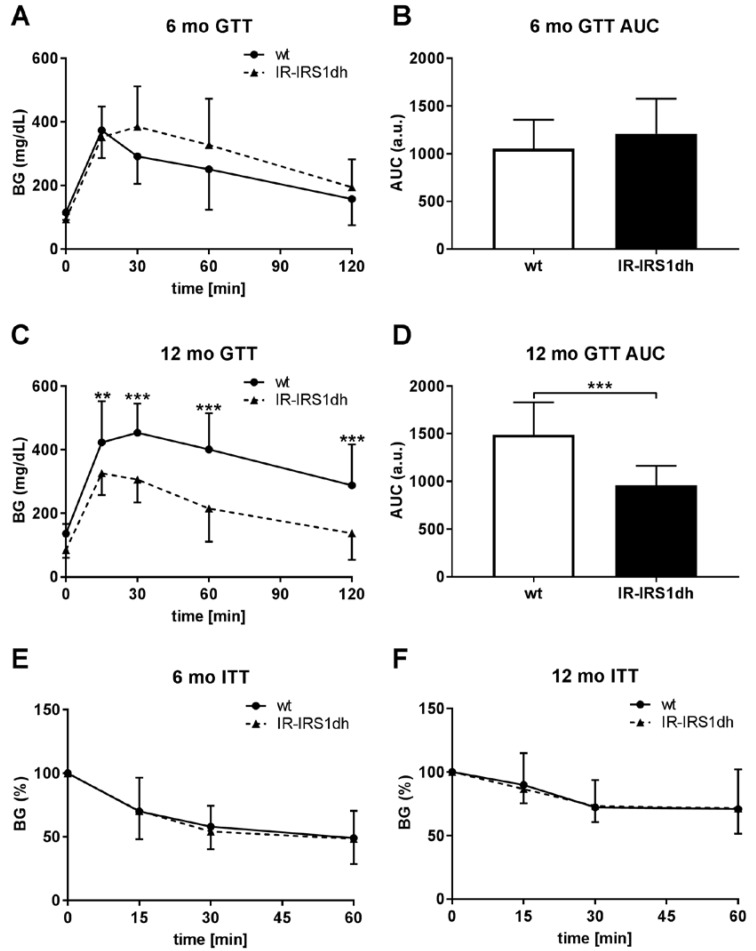
Glucose and insulin tolerance of IR-IRS1dh mice. (**A**,**C**) Blood glucose (BG) levels during intraperitoneal glucose tolerance tests (GTT) and (**B**,**D**) area under the curve (AUC) of GTT results. (**E**,**F**) Blood glucose levels during insulin tolerance tests (ITT) normalized to 0 min blood glucose levels (100%). Columns and symbols represent mean values ± standard deviation; (**A**–**D**) *n*: 11–24, (**E**,**F**) *n*: 5–13; * denotes significant differences between IR-IRS1dh mice compared to wt controls; ** *p* < 0.01, *** *p* < 0.001.

Furthermore, insulin was elevated, but insulin-like growth factor 1 (IGF-1) remained unchanged in IR-IRS1dh mice (Table 1). Serum leptin level was lower in 12-month-old IR-IRS1dh mice compared to age-matched controls (Table 1). To study glucose metabolism, we performed glucose and insulin tolerance tests. While in 6-month-old IR-IRS1dh mice there seemed to be a tendency of impaired glucose tolerance, 12-month-old IR-IRS1dh mice demonstrated improved glucose metabolism compared to age-matched wt controls (Figure 1A–D).

Insulin tolerance was normal in IR-IRS1dh mice in both ages (Figure 1E–F). These results indicate that the ablation of one copy of IR and IRS-1 caused a compensatory hyperinsulinemia. The improved glucose tolerance comparing 6 to 12 months of age suggests an adaptive mechanism in IR-IRS1dh mice protecting against an ageing-induced defect of glucose metabolism.

### 2.2. Mitochondrial Performance in Skeletal Muscle of IR-IRS1dh Mice

**Figure 2:**
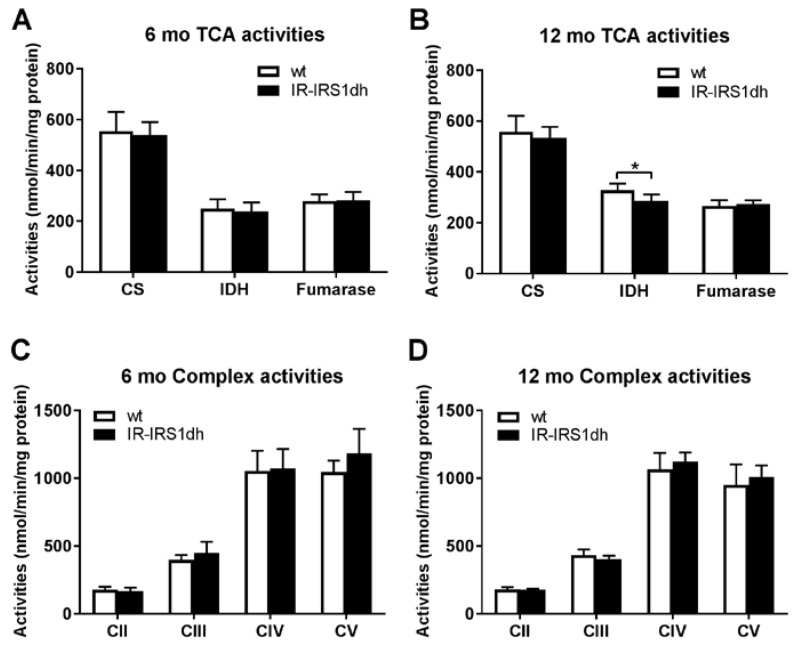
Skeletal muscle mitochondrial activities of IR-IRS1dh mice. (**A**,**B**) Mitochondrial tricarboxylic cycle (TCA) and (**C**,**D**) respiratory complex activities determined in skeletal muscle homogenates. CS: citrate synthase; IDH: isocitrate dehydrogenase; C denotes mitochondrial complexes II–V. Columns represent mean values ± standard deviations, *n*: 4–6; * denotes significant differences between IR-IRS1dh mice compared to wt controls; * *p* < 0.05.

In order to investigate muscle mitochondrial function, activities of TCA cycle enzymes as well as individual respiratory chain complexes were measured in muscle homogenates. Mitochondrial enzyme activities were rather normal in IR-IRS1dh mice (Figure 2).

**Figure 3:**
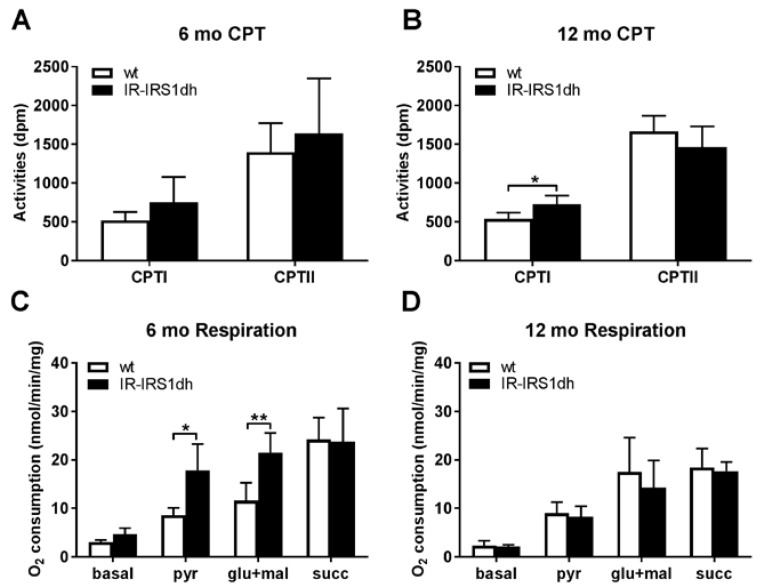
Skeletal muscle mitochondrial performance of IR-IRS1dh mice. (**A**,**B**) Carnitine palmitoyl transferase activities (CPT I and II) measured in skeletal muscle homogenates. (**C**,**D**) Mitochondrial respiration determined in soleus muscle. pyr: pyruvate; glu: glutamate; mal: malate; succ: succinate. Pyruvate and glutamate were used as complex I substrates, succinate as complex II substrate. Columns represent mean values ± standard deviation; *n*: 4–6; * denotes significant differences between IR-IRS1dh mice compared to wt controls; * *p* < 0.05, ** *p* < 0.01.

The activity of carnitine palmitoyl transferase I (CPTI), which is the rate limiting enzyme for transporting fatty acids into mitochondria, tended to be higher in 6-month-old IR-IRS1dh mice and was significantly higher in 12-month-old IR-IRS1dh mice compared to aged-matched controls (Figure 3,B).

In situ skeletal muscle mitochondrial performance was assessed by high resolution respirometry and 6-month-old IR-IRS1dh mice showed elevated complex I driven respiration (Figure 3C), however, mitochondrial performance in 12-month-old IR-IRS1dh mice was unchanged (Figure 3D). These results indicate that the deficiency in IR and IRS1 resulted in higher skeletal muscle mitochondrial functional activity at 6 months of age and was reverted to normal levels during ageing.

### 2.3. Mitochondrial Performance in Liver of IR-IRS1dh Mice

**Figure 4:**
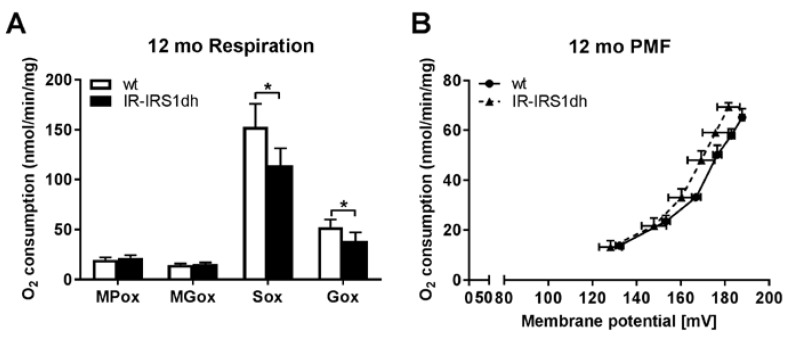
Liver mitochondrial performance of IR-IRS1dh mice. (**A**) Liver mitochondrial respiration determined by polarography. MPox: malate and pyruvate oxidation; MGox: malate and glutamate oxidation; Sox: succinate oxidation; Gox: glyceraldehyde 3-phosphate oxidation. Pyruvate and glutamate were used as complex I, succinate as complex II, and glyceraldehyde 3-phosphate as complex III substrates, respectively. (**B**) Liver mitochondrial proton motive force (PMF) measurements. Columns and symbols represent (**A**) mean values ± standard deviation or (**B**) mean values ± standard error; *n*: 5, * denotes significant differences between IR-IRS1dh mice compared to wt controls; * *p* < 0.05.

In order to investigate liver mitochondrial function, we measured oxygen consumption and mitochondrial membrane potential in isolated liver mitochondria. We found a lower complex II and III driven mitochondrial respiration in IR-IRS1dh mice at both ages compared to wt controls (as described previously [5] and Figure 4A).

We also observed a significant decrease in mitochondrial membrane potential in 6-month-old IR-IRS1dh mice compared to wt controls, as described previously [5], while 12-month-old IR-IRS1dh mice only showed a tendency of reduced mitochondrial membrane potential (Figure 4B). These results suggest that ablation of one copy of IR and IRS1 decreased hepatic mitochondrial performance, which was not reverted upon ageing, in contrast to muscle.

## 3. Discussion

IR^+/−^-IRS-1^+/−^ double heterozygous (IR-IRS1dh) mice showed attenuated insulin signaling in skeletal muscle and liver [12], which caused a pancreatic β-cell hyperplasia [9,11], in parallel with the elevated insulin levels (Table 1). These data suggest that the overproduction of insulin could be a compensatory mechanism counteracting peripheral insulin resistance in IR-IRS1dh mice, which also led to lower fasting blood glucose levels [9]. Since ablation of both copies of IR in the liver (LIRKO mice) also results in fasting hypoglycemia at 6 months of age despite random fed hyperglycemia, it is conceivable that fasting blood glucose levels are independently regulated from postprandial glucose levels upon ablating insulin receptor in the liver [13]. Six-month-old IR-IRS1dh mice exhibited a tendency of impaired glucose tolerance, which is in line with the findings of Kulkarni et al. [14]. Interestingly, 12-month-old IR-IRS1dh mice demonstrated improved glucose tolerance compared to age-matched wt controls. Furthermore, 12-month-old IR-IRS1dh mice also showed a 25% lower body weight, which could be partly attributed to lower white adipose tissue weight (data not shown). These results are in line with the lower leptin levels observed in these animals. Since body weight changes are known to influence energy metabolism [15], the lower body weight and/or lower white adipose tissue found in 12-month-old IR-IRS1dh mice could also contribute to improved glucose tolerance. Similar to our findings, LIRKO mice also showed improved glucose tolerance during ageing (impaired glucose tolerance at 2 months of age and normal glucose tolerance at 6 months of age) [13], suggesting that insulin receptor signaling is altered during ageing as an adaptation. These results suggest that at 12 months of age, wt mice of this strain spontaneously developed an ageing-induced impaired glucose tolerance, while IR-IRS1dh mice were protected against this detrimental effect of ageing. There is indeed an increasing number of studies which suggest that moderately impaired insulin signaling is associated with beneficial metabolic effects during ageing, leading to increased longevity in worms, flies, and mice [16]. Further studies have suggested that the increased longevity in mice with impaired insulin signaling is mainly due to insulin effects on brain and fat tissues [17,18]. Interestingly, the ablation of insulin receptor in white adipose tissue (FIRKO mice) also resulted in an improved metabolic state during ageing, since FIRKO mice were protected against ageing-induced impaired glucose tolerance [19]. In summary, these results indicate that attenuated insulin signaling in 12-month-old IR-IRS1dh mice possibly plays a role in the improved glucose metabolism.

Skeletal muscle mitochondrial dysfunction was shown to be associated with muscle insulin resistance in humans and mice [4,20,21] and decreased mitochondrial performance is postulated to be an adaptation to nutrient induced changes in energy expenditure during insulin resistance [22]. Insulin deprivation was shown to decrease mitochondrial ATP production in patients with type 1 diabetes [23] and insulin treatment of human or rat muscle cells in vitro elevated oxygen consumption and respiratory control ratio [24]. These data suggest that insulin could directly regulate mitochondrial function in skeletal muscle. IR-IRS1dh mice demonstrated higher insulin levels, which may be involved in stimulation of the increased skeletal muscle respiration found in 6-month-old animals. On the other hand, 12-month-old mice still showed hyperinsulinemia but normal mitochondrial function in skeletal muscle, therefore other factors in addition to insulin have to be involved in the increased mitochondrial performance observed in 6-month-old IR-IRS1dh animals. Whether mitochondrial dysfunction is a cause or consequence of insulin resistance has not been resolved yet, but our results of IR-IRS1dh mice showing insulin resistance but elevated (at 6 months of age) or normal (at 12 month of age) muscle mitochondrial function suggest that mitochondrial dysfunction is not a self-evident process in insulin resistant condition.

The interaction between insulin signaling and mitochondrial function in the liver is even less understood [25]. Reduced, elevated, or normal mitochondrial function was found in animal models or humans with non-alcoholic fatty liver disease (NAFLD)/non-alcoholic steatohepatitis (NASH) [5,26,27]. The current data suggest that in an early disease state of increased hepatic lipid levels (like NAFLD), mitochondrial function is elevated probably via a compensatory mechanism [6], however, in the later disease stages with inflammation (like NASH), mitochondrial function is reduced [28]. In IR-IRS1dh mice, the defect in insulin signaling led to a clear reduction of mitochondrial performance in both ages, which was also associated with decreased mitochondrial membrane potential at 6 months of age. These results suggest that insulin signaling is tightly interconnected with mitochondrial function, but the detailed biological pathways behind the cross-talk are not resolved yet [29]. Insulin resistant ob/ob mice were characterized by impaired hepatic mitochondrial function and altered mitochondria-associated endoplasmic reticulum (ER) membranes (MAM) morphology [30]. Furthermore, increasing the defective coupling between ER and mitochondria improved insulin signaling in vitro [31]. Therefore, MAM could play an important role in hepatic insulin action and mitochondrial function/dynamics. Recent studies indicate that several key insulin signaling molecules like AKT and mTORC2 are localized in MAM and they could serve as an interaction site between insulin signaling and mitochondrial function [29]. Another possible factor that could also influence hepatic mitochondrial function in IR-IRS1dh mice is the altered body weight. Obese rodent models fed with high fat diet demonstrate elevated mitochondrial respiration in skeletal muscle and liver [5,32]. Furthermore, obese patients also showed increased hepatic mitochondrial respiration compared to lean controls [26]. Therefore, the reduced mitochondrial respiration observed in IR-IRS1dh mice may be partly attributed to lower body weight.

To overcome mitochondrial dysfunction in metabolic diseases, mitochondrial targeting drugs were applied, which show beneficial metabolic effects [33]. Recently, we and others have shown that peroxisome proliferator-activated receptor (PPAR) and PPAR gamma coactivator 1-α (PGC-1α) activating drugs improve mitochondrial mass/function and, in turn, ameliorate insulin resistance and diabetes [34,35,36]. These results suggest that mitochondrial dysfunction is a crucial metabolic disturbance in insulin resistance, and targeting mitochondria is a promising approach for the treatment of diabetes.

## 4. Materials and Methods

### 4.1. Animal Studies

Since metabolic phenotype was previously shown to be more profound in male mice [14], only male animals were used in our study. Mice received a standard diet containing 16.4% protein, 4% fat, and 48.5% carbohydrates (Harlan/Envigo, Indianapolis, IN, USA) and had ad libitum access to food and water. IR-IRS1dh mice were backcrossed to C57BL/6N background for eight generations. All animals received humane care and mouse studies were approved by Landesamt für Natur, Umwelt und Verbraucherschutz Nordrhein-Westfalen (2010, Reference number 20.11.245) and performed according to GV-SOLAS (Society for Laboratory Animal Science) in accordance with the German Animal Welfare Act. Dissected tissues were prepared as stated below. Blood glucose levels were measured in tail blood samples using a glucometer (A. Menarini Diagnostics, Berlin, Germany) and serum insulin, IGF-1, and leptin levels were determined with ELISA (Chrystal Chem Inc., Downers Grove, IL, USA and R&D Systems Minneapolis, MN, USA). Intraperitoneal glucose tolerance tests were performed with 2 g/kg glucose and intraperitoneal insulin tolerance tests were performed with 0.75 U/kg insulin.

### 4.2. Mitochondrial Enzyme Activities

Musculus soleus and plantaris (red muscles) were dissected and frozen in liquid nitrogen. Muscles were homogenized in homogenization buffer (255 mM sucrose, 2 mM ethylene glycol-bis(β-aminoethyl ether)-tetraacetic acid (EGTA), 40 mM KCl, 0.1 % bovine serum albumin, 20 mM Tris, pH 7.2). Mitochondrial complex (CII-CV) and TCA enzyme (citrate synthase, isocitrate dehydrogenase, fumarase) activities as well as carnitine palmitoyl transferase activities (CPT I and II) were determined in muscle homogenates, as described previously [4].

### 4.3. High Resolution Respirometry

Contralateral soleus muscles were used immediately for oxygen consumption studies, as described previously [4]. Briefly, isolated muscle fibers were saponin treated and analyzed using high resolution respirometry (Oroboros Instruments, Inssbruck, Austria) at the following conditions. Basal respiration (basal): 5 mM MgCl_2_, 10 mM pyruvate, 0.5 mM malate. Pyruvate respiration (pyr): 2 mM ADP. Glutamate and malate respiration (glu + mal): 10 mM glutamate, 10 mM malate. Succinate respiration (succ): 10 mM succinate, 0.5 μM rotenone.

### 4.4. Polarography

Briefly, liver mitochondria were freshly isolated from liver by differential centrifugation and resuspended in homogenization buffer (see above). Oxygen consumption was determined with Clark-electrodes (Hansatech Instruments, Norfolk, UK) at the following conditions, as described previously [37]. Malate and pyruvate oxidation (MPox): 0.2 mM malate, 8 mM pyruvate, 0.4 mM ADP, 1 mM NAD^+^. Malate and glutamate oxidation (MGox): 10 mM malate, 10 mM glutamate. Succinate oxidation (Sox): 10 mM succinate, 0.4 mM ADP, 4 µM rotenone. Glyceraldehyde 3-phosphate oxidation (Gox): 10 mM glyceraldehyde 3-phosphate.

### 4.5. Proton Motive Force Measurement

Briefly, liver mitochondria were freshly isolated from liver during differential centrifugation and resuspended in homogenization buffer (see above). Mitochondrial membrane potential was determined using a methyltriphenylphosphonium (TPMP) ion-sensitive electrode, while analyzing in parallel the respiration rate with a Clark type electrode (Hansatech Instruments, Norfolk, UK) in a proton leak titration assay, as described previously [5,38].

### 4.6. Statistics

Statistical evaluations were performed using GraphPad Prism 7.02 (GraphPad Software Inc, La Jolla, CA, USA). Two tailed, unpaired Student’s *t*-tests were applied with unequal distribution when two groups were compared and two-way analysis of variance (ANOVA) with post hoc Holm-Sídák’s multiple comparison tests were used to calculate statistical significance for GTT and ITT results comparing different time points and groups. Statistical significance was assumed at *p* < 0.05.

## Figures and Tables

**Table 1:** Metabolic parameters.

Mouse Parameters	6 mo wt	6 mo IR-IRS1dh	12 mo wt	12 mo IR-IRS1dh
BW (g)	33.4 ± 5.9 (11)	28.0 ± 1.7 * (24)	39.2 ± 6.0 (11)	29.7 ± 1.9 *** (20)
Fasting BG (mg/dL)	115.8 ± 23.7 (11)	93.8 ± 16.4 * (24)	135.8 ± 30.3 (11)	84.8 ± 24.7 *** (20)
Insulin (pg/mL)	664 ± 565 (11)	2325 ± 1455 *** (22)	2891 ± 1656 (8)	5390 ± 1454 ** (15)
Leptin (pg/mL)	3138 ± 1589 (14)	2314 ± 1340 (22)	5585 ± 853 (7)	2363 ± 685 *** (15)
IGF-1 (pg/mL)	534 ± 142 (14)	543 ± 160 (23)	631 ± 133 (8)	587 ± 186 (12)

Body weight (BW), fasting blood glucose (BG), random fed serum insulin, leptin, and insulin-like growth factor 1 (IGF-1) levels, mo denotes the age of mice in months. Numbers represent mean values ± standard deviation, numbers in parenthesis indicate numbers of biological replicates, * denotes significant differences between IR-IRS1dh mice compared to age-matched wild-type (wt) controls; * *p* < 0.05, ** *p* < 0.01, *** *p* < 0.001. Part of these data have been published previously [5].

## Abbreviations

wt	wild-type
IR	insulin receptor
IRS-1	insulin receptor substrate-1
IR-IRS1dh mice	insulin receptor (IR)^+/–^-insulin receptor substrate-1 (IRS-1)^+/–^ double heterozygous mice
mo	months
TCA	tricarboxylic cycle
KO	knockout
NAFLD	non-alcoholic fatty liver disease
NASH	non-alcoholic steatohepatitis
PPAR	peroxisome proliferator-activated receptor
PGC1α	peroxisome proliferator-activated receptor gamma coactivator 1-alpha
